# Multimodal Management of Grade 1 and 2 Pancreatic Neuroendocrine Tumors

**DOI:** 10.3390/cancers14020433

**Published:** 2022-01-15

**Authors:** Ugo Marchese, Martin Gaillard, Anna Pellat, Stylianos Tzedakis, Einas Abou Ali, Anthony Dohan, Maxime Barat, Philippe Soyer, David Fuks, Romain Coriat

**Affiliations:** 1 Department of Digestive, Hepatobiliary and Pancreatic Surgery, Cochin Teaching Hospital, AP-HP, Université de Paris, 27 rue du Faubourg Saint-Jacques, 75014 Paris, France; martin.gaillard@aphp.fr (M.G.); stylianos.tzedakis@aphp.fr (S.T.); david.fuks@aphp.fr (D.F.); 2 Gastroenterology and Digestive Oncology Unit, Cochin Teaching Hospital, AP-HP, Université de Paris, 27 rue du Faubourg Saint-Jacques, 75014 Paris, France; anna.pellat@aphp.fr (A.P.); einas.abouali@aphp.fr (E.A.A.); romain.coriat@aphp.fr (R.C.); 3 Department of Radiology, Cochin Teaching Hospital, AP-HP, Université de Paris, 27 rue du Faubourg Saint-Jacques, 75014 Paris, France; Anthony.dohan@aphp.fr (A.D.); maxime.barat@aphp.fr (M.B.); philippe.soyer@aphp.fr (P.S.)

**Keywords:** pancreatic neuroendocrine tumor, pancreatectomy, pancreatic sparing surgery, enucleation, chemotherapy, therapeutic strategy

## Abstract

**Simple Summary:**

As we are facing an increasing incidence of pancreatic neuroendocrine tumors (pNETs), it appears necessary to better classify this disease—and in 2017 the WHO classification introduced a new category of well differentiated grade 3 tumors. pNETs treatment requires some specific background and recent reviews on the multimodal management of this disease are missing. Indeed, those modalities constantly evolve and this review, focusing on Grade 1 and Grade 2 pNETs, aims to clarify both oncological and surgical treatment options from localized tumors to multi metastatic disease. Every aspect of the strategy are discussed in this review from the oncologist and the surgeon’s point of view and with a special focus on a minimally invasive approach.

**Abstract:**

Pancreatic neuroendocrine tumors (p-NETs) are rare tumors with a recent growing incidence. In the 2017 WHO classification, p-NETs are classified into well-differentiated (i.e., p-NETs grade 1 to 3) and poorly differentiated neuroendocrine carcinomas (i.e., p-NECs). P-NETs G1 and G2 are often non-functioning tumors, of which the prognosis depends on the metastatic status. In the localized setting, p-NETs should be surgically managed, as no benefit for adjuvant chemotherapy has been demonstrated. Parenchymal sparing resection, including both duodenum and pancreas, are safe procedures in selected patients with reduced endocrine and exocrine long-term dysfunction. When the p-NET is benign or borderline malignant, this surgical option is associated with low rates of severe postoperative morbidity and in-hospital mortality. This narrative review offers comments, tips, and tricks from reviewing the available literature on these different options in order to clarify their indications. We also sum up the overall current data on p-NETs G1 and G2 management.

## 1. Introduction, Epidemiology and Staging

Neuroendocrine neoplasms (NENs) originate from neuroendocrine cells present throughout the endocrine system. Pancreatic neuroendocrine tumors (p-NETs) arise from pancreatic stem cells that can differentiate into exocrine and endocrine but also directly from neuroendocrine cells in the pancreas [1,2]. p-NETs were previously regarded as rare, but their incidence has increased over the last 20 years [3]. A Japanese study published in 2005 showed a high incidence of p-NETs (1.01 per 100,000) [4] compared to incidence usually considered in the United States (0.3–0.4 per 100,000) [5]. Peak age ranges from 50 to 70 years, and incidence increases with age.

Most p-NETs are non-functioning and symptoms usually stem from distant metastases or sometimes from mass effects [6,7].

In 2017, the World Health Organization (WHO) updated the grading system for the pathological classification of p-NENs, based on the Ki-67 index and mitotic counts [2]. Tumors with a Ki-67 index of ≤2%, 3–20% or >20% were classified as G1, G2 and G3, respectively. Until 2017, pancreatic carcinoid neoplasms were defined as G1/G2 p-NETs, while pancreatic neuroendocrine carcinoma (p-NEC) was defined as G3 (Table 1). The 2017 classification introduced a new entity of p-NETs, the p-NETs G3 [8]. While this group of well-differentiated G3 p-NETs respond poorly to platinum-based chemotherapy compared to low differentiated G3 p-NEC, they are associated with longer median survival [9]. This group of G3 p-NETs should be distinguished from G1/G2 p-NETs and will not be discussed in the present review.

The 5-year survival rate of patients with p-NETs is estimated to be 85% for all stages, 65–100% in localized, 35% in regional and 20% in metastatic diseases. The 5-year survival rate of patients increases when managed in dedicated centers [10]. According to the Surveillance, Epidemiology, and End Results (SEER) database, 53% of patients with NETs present with localized disease, 20% have locoregional disease, and 27% have distant metastases at the time of diagnosis [5]. Individuals with a family history of NETs in a first-degree relative have a 3.6-fold increased risk of disease [11]. No environmental risk factors have been identified yet.

Preoperative endoscopic ultrasonography with biopsy for histopathological confirmation is mandatory. Preoperative staging should also include morphological evaluation and, whenever possible, functional evaluation by ^68^gallium-positron emission tomography/CT (PET/CT). However, all tumors do not express a significant number of somatostatin type 2 receptors (SSTR). Therefore, DOTA-TOC PET/CT should always be associated with preoperative CT or magnetic resonance imaging (MRI). PET/CT with specific tracers such as 11C-5HTP, 18F-DOPA or 18F-FDG can further optimize the staging of the disease [12,13,14]. Endoscopy associated with biochemical analysis of relevant biomarkers such as pCgA are often helpful in these situations.

Approximately 10% of p-NETs are due to an inherited syndrome, which includes multiple endocrine neoplasia type 1 (MEN1), von Hippel-Lindau disease (VHL), neurofibromatosis type 1 (NF1), and tuberous sclerosis complex (TSC) [15]. In patients with sporadic p-NETs, 20 to 40% express somatic mutation of *MEN1* or other specific mutations such as *DAXX*, *ATRX* and *TSC2* [16,17]. 

## 2. Multimodal Management of Localized Disease

### 2.1. Indications

Localized NETs’ treatment strategy depends on tumor diameter, tumor location, and patient condition. If feasible, surgery is the best option and is often the best treatment for most localized p-NETs, except for incidental lesions. Indeed, the incidence of early p-NETs has increased significantly [18]. Several series and a review published by Partelli S et al. in 2017 [19] suggested that patients with incidental tumors less than 2 cm in diameter experienced a limited risk of tumor recurrence and were all alive at 5 years. The European Neuroendocrine Tumor Society (ENETS) guidelines then proposed a “watch and wait” policy in asymptomatic patients with sporadic < 2 cm low-grade (G1) p-NETs [20]. Histopathological confirmation is mandatory in this situation [21], but no consensus has emerged regarding the follow-up protocol. A first computed tomography (CT) or magnetic resonance imaging (MRI) examination at 6 months from diagnosis followed by CT or MRI examination every year seems to be a safe option.

Surgery including either standard or atypical pancreatic resections represents the gold standard for p-NETs > 2 cm in symptomatic forms in the case of functional tumors and/or in G2 tumors. Carcinoid syndrome cannot appear in localized form as it is related to serotonin secretion from liver metastases (LM) but not from the primary pancreatic tumor. Indeed, the hormones released into the portal vessels are metabolized by the liver. Lymphadenectomy’s impact on patients’ oncological outcomes is still unclear [22]. The rate of mortality after pancreatic standard resections in high volume centers remains acceptable (<5%), even if the postoperative complication rates remain high (40–50%) [23,24]. These radical pancreatectomies are associated with a significant rate of long-term exocrine and endocrine insufficiencies [25]. There is no place for adjuvant chemotherapy in this setting.

### 2.2. Minimally Invasive Approach

In 1994, the first descriptions of minimally invasive pancreatic procedures were reported, consisting of laparoscopic pylorus-preserving pancreatoduodenectomy and laparoscopic pancreatic distal resection [26].

Because of the rarity of p-NETs, no specific comparative studies have reported the perioperative outcomes of minimally invasive surgery in this indication. A review by Drymousis et al. including 11 studies published between 1994 and 2012 on laparoscopic and open pancreatic procedures in 906 patients with p-NETs reported that in the laparoscopic group, there was a reduced overall complication rate and length of stay but no differences in mortality, operative time or pancreatic fistula rates [27]. Only two patients undergoing pancreaticoduodenectomy were included, while most of the resections were distal pancreatectomy and enucleation. Most series reported a 10 to 40% conversion rate.

From the perspective of pancreatic adenocarcinoma and given oncological results, including surgical margins and harvested lymph nodes, the laparoscopic approach in distal pancreatectomy (LDP) allows for similar results with an open approach [28]. In a nationwide analysis, De Rooij et al. showed the superiority of the LDP for benign and malignant pancreatic tumors regarding the occurrence of major complications and the length of hospital stay [29]. The same group conducted a prospective controlled randomized trial to compare LDP to the open procedure (LEOPARD-Trial) that demonstrated the benefits of the minimally invasive approach [30]. However, the LEOPARD trial for distal pancreatectomy included not only p-NETs but also other pancreatic diseases such as adenocarcinoma.

Standard pancreatoduodenectomy remains necessary when enucleation cannot be considered. Laparoscopic pancreaticoduodenectomy (LPD) has seen much slower adoption than other laparoscopic procedures as it represents a true technical challenge, especially when it comes to reconstruction. Concerns remains regarding the long learning curve of LPD, potential higher morbidity and mortality rates, and the need to perform a high number of procedures (more than 40 procedures) to achieve equivalent outcomes to the open approach [31]. Since 2017, three trials and one meta-analysis have compared the short-term outcomes of LPDc versus open pancreaticoduodenectomy [32,33,34]. No differences were observed, except for lower blood loss and longer operative time with the laparoscopic approach.

Recently, the multicenter randomized LEOPARD-2 trial had to be stopped prematurely because of safety concerns regarding a higher mortality rate after LPD, although overall 90-day mortality (five of 50 patients (10%) in the LPD group vs. one of 49 (2%) in the open pancreatoduodenectomy group; risk ratio (RR): 4.90 (95% CI: 0.59–40.44); *p* = 0.20) and severe complication rate (14 (28%) in the LPD group vs. 12 (24%) in the open pancreatoduodenectomy group; RR: 1.14 (95% CI: 0.59–2.22); *p* = 0.69) were not significantly different [35]. In light of these results, LPD seems to remain a technical step too complex to allow for the safe diffusion of this technique. In this setting, the French Health Agency has recently both promoted the LDP and banned LPD outside a prospective trial inclusion. There is a high probability that several national health agencies will take a similar decision if no series invalidate these disappointing results.

Finally, total pancreaticoduodenectomy could be an option for multifocal p-NETs as they might occur in patients with MEN1 and VHL syndrome. In these selected patients, laparoscopic total pancreatectomy has been successfully performed [36]. However, there are no data comparing laparoscopic to open total pancreatectomy in p-NETs.

### 2.3. Robotic Approach

The first robotic-assisted distal pancreatectomy for the treatment of p-NETs was reported in 2002 using the DaVinci^®^ (Intuitive Surgical, Sunnyvale, CA, USA) robotic surgical system [37]. Robotic assistance was helpful to increase the spleen preservation rate in distal pancreatectomy due to better depth perception owing to the use of three-dimensional representation, tremor elimination and seven degrees freedom of movement [38,39].

In a retrospective study, Zhang et al. compared 43 patients who underwent robotic distal pancreatectomy with 31 patients who underwent laparoscopic distal pancreatectomy for the treatment of p-NETs [40]. Again, a higher rate of spleen preservation was observed in the robotic group (79.1% vs. 48.4%; *p* = 0.006) as well as a number of lymph nodes harvested for G2 and G3 p-NETs (3.5 vs. 2; *p* = 0.034). Daouadi et al. also confirmed these findings in a retrospective analysis of 124 patients and noticed a reduced conversion rate in robotic-assisted distal pancreatectomy (0% vs. 16% in the laparoscopic approach; *p* < 0.05) and reduced operating time (293 ± 93 (SD) min vs. 372 ± 141 (SD) min; *p* < 0.01) [41]. On the contrary, a longer robot operative time was observed by Ryan et al. when compared with the laparoscopic approach (221.4 vs. 173.6 min; *p* = 0.026) in a prospective observational study [42], and similar results were reported by Lai et al. [43]. However, when splenectomy is mandatory, robotic assistance has not demonstrated advantages for distal pancreatectomy [44].

Even if the experience remains limited, a few centers have reported a perioperative mortality rate from 0 to 5% and a pancreatic fistula rate from 0 to 35% after robotic-assisted pancreatoduodenectomy [45,46]. These results are similar to those obtained with open surgery. Interestingly, the learning curve was significantly improved after the first 20 performed procedures; however, improvement concerning pancreatic fistula appears only after 40 performed procedures and operative time after 80 procedures [46]. Conversion rate after completion of the learning curve was 3.3%, mortality was 3.3% and grade B/C pancreatic fistula rate was 6.9%.

### 2.4. Enucleation

Patients with functional p-NETs usually have small tumors at the time of diagnosis, particularly pancreatic insulinomas, which are benign in 80–90% of patients. Considering the usually small size of tumors at diagnosis, parenchyma-preserving limited pancreatic resection such as pancreatic enucleation should be considered [47,48]. According to guidelines, pancreatic enucleation is an excellent option if the tumor is exophytic or located close to the surface and with a minimum distance of 2–3 mm to the main pancreatic duct [49].

Concerning nonfunctional pNETs: on the one hand, non-functional p-NETs are supposed to present more aggressive behavior, especially tumors larger than 2 cm, and radical resection with lymphadenectomy should be performed [17]. On the other hand, many recent data support the feasibility of the “watch and wait” policy for asymptomatic nonfunctional p-NETs < 2 cm as it was first proposed in MEN1 patients. Indication of enucleation is unclear and even if pancreatic enucleation allows us to spare the pancreatic parenchyma and function, it has not proven its positive impact on postoperative outcomes and should not be proposed as an alternative to radical resection for patients who are candidates for surgery.

However, the distinction between benign and malignant p-NETs based on tumor diameter should be drawn cautiously, as even small tumors can be aggressive with lymph node involvement [49]. Conversely, some groups have claimed that local lymphadenectomy should not be considered routinely, even for G1 non-functional p-NETs [50] since no clear oncological benefit is demonstrated.

Minimally invasive experience in pancreatic enucleation included mostly small series. Along with a retrospective series of 15 laparoscopic pancreatic enucleations and 22 open pancreatic enucleations, Zhang et al. observed similar mortality and morbidity. Up to 20% of the patient in the laparoscopic group had a pancreatic fistula grade B/C versus 36.4% in the open group (*p* = 0.874) [51]. They also observed a significantly shorter operating time (118.2 ± 33.1 (SD) min vs. 155.2 ± 44.3 (SD) min; *p* = 0.004), lower estimated blood loss (80.0 ± 71.2 (SD) mL vs. 195.5 ± 103.4 (SD) mL; *p* = 0.001), shorter first flatus time (1.8 ± 1.0 (SD) days vs. 3.4 ± 1.8 (SD) days; *p* = 0.004) and shorter hospital stay (2.4 ± 1.0 (SD) vs. 4.4 ± 2.0 (SD) days; *p* = 0.001). During the follow-up (median 47 months), no local recurrence or distant metastases occurred in either group [52]. It should be noted that there is a relevant risk for pancreatic fistula after pancreatic enucleation. Karaliotas et al. showed similar pancreatic fistula rates in open group and in laparoscopic group pancreatic enucleation (29% vs. 20%, respectively; *p* = 0.65) [53] but the pancreatic fistula rate was lower in the laparoscopic group in Sa Cunha et al.’s series (14% vs. 100%, respectively; *p* = 0.015) [54]. Therefore, the superiority of laparoscopic pancreatic enucleation should be confirmed by further trials.

### 2.5. Non-Sporadic p-NETs

Indication of surgery for nonfunctional pNETs is conditioned mostly by the size of the lesion > 2 cm [55]. The same strategy can be proposed for gastrinoma but regardless of their size, the resection of insulinomas, VIPomas, and glucagonomas is recommended [56]. In the particular setting of MEN1, virtually all patients may present with multiple lesions and additional non-functional p-NETs and it can be challenging because of possible difficulties in identifying multiple tumors within the pancreatic parenchyma. Spleen preserving distal pancreatectomy or pancreatic enucleation is a successful approach in 83 to 100% of patients with a single p-NET [57,58].

P-NETs also occur in patients with VHL disease, a hereditary, multi-organ tumor disease, with hemangioblastomas, pheochromocytomas and renal clear cell carcinomas [59]. P-NETs are found in 8% to 17% of patients with VHL disease. They are usually non-functional but should be distinguished from cystic benign pancreatic lesions that are frequent in this disease. They are considered less invasive than non-functional p-NETs without VHL because they are usually discovered before symptoms appear, during surveillance of VHL disease [60]. A cut-off of 30 mm or more is used to decide on surgical resection given the risk of metastases [61]. 

## 3. Multimodal Strategy for Advanced p-NETs

### 3.1. Medical Treatment Options

Medical treatment can either be proposed in the neoadjuvant setting for patients with important initial tumor burden or for unresectable advanced p-NETs. In the meantime, no clear data support the medical treatment of p-NETs in a neoadjuvant setting and this strategy should be considered cautiously. Several types of treatments are currently available and the choice of molecule needs to consider the patient’s general state as well as the tumor’s aggressiveness.

### 3.2. Cytotoxic Chemotherapy

Chemotherapy is the usual first-line treatment in aggressive metastatic p-NETs with important hepatic tumor burden. Various combinations of chemotherapy are validated in G1/G2 p-NETs. Streptozocin (SPTZ) combined to fluorouracil and doxorubicine (DOX) showed positive results in the treatment of well-differentiated p-NETs with a response rate of 39% [62]. Several studies have also shown the efficacy of SPTZ associated with 5-fluoruracil (5-FU) or DOX [63,64,65]. Because of toxicity, the combination of SPTZ and 5-FU is now favored. The combination of temozolomide and capecitabine (TEMCAP) showed a 70% response rate and a median progression-free survival (PFS) of 18 months in a population of 30 patients [66]. Preliminary results of a recent phase II trial involving 144 patients with p-NETs comparing temozolomide alone to TEMCAP showed improved OS and PFS with TEMCAP [67]. A retrospective analysis comparing TEMCAP to 5-FU and dacarbazine (DTIC) suggested longer PFS with 5-FU-DTIC, although the TEMCAP regimen was often preferred due to its oral administration [68]. Finally, an oxaliplatin-based regimen can be proposed as an alternative based on interesting results in small populations of p-NETs [69,70].

There is little data regarding the efficacy of chemotherapy in the second-line setting. One single-center study found a 17% response rate in 18 patients with progressive disease treated with gemcitabine and oxaliplatin (GEMOX) [71]. In clinical practice, previous regimens are also used in the second-line setting in the case of progressive disease under another type of treatment.

### 3.3. Somatostatin Analogs (SSA)

SSA are often used to control p-NET-related symptoms (secretory syndrome). The phase III CLARINET trial comparing lanreotide vs. placebo in metastatic nonfunctional G1/G2 enteropancreatic NET validated SSA efficacy in these patients. Lanreotide was associated with significantly prolonged PFS (median not reached vs. median of 18 months, *p* < 0.001; hazard ratio (HR) for progression or death of 0.47; 95% CI: 0.30–0.73) [72]. SSA are mainly proposed in the first-line setting of advanced p-NETs with low hepatic tumor burden and slow disease progression.

### 3.4. Targeted Therapy

A number of targeted therapeutic agents, especially those inhibiting molecules involving angiogenesis or growth factor receptor-related signal pathways have been evaluated in p-NETs [73,74]. Sunitinib and everolimus are currently the two drugs approved in both the USA and Europe to treat patients with well-differentiated p-NETs in the second-line setting.

#### 3.4.1. Agents for Antiangiogenesis

P-NETs are hypervascular tumors associated with intense expression of vascular endothelial growth factor (VEGF), VEGF receptor (VEGFR) and platelet derived growth factor receptor (PDGFR) [75,76,77].

Sunitinib is a tyrosine kinase inhibitor (TKI), which targets multiple receptors such as PDGFR and VEGFR [78]. In 2011, a phase III randomized controlled trial confirmed that sunitinib was efficient at increasing PFS (11.4 months vs. 5.5 months; *p* < 0.001) and objective response rates (9.3% vs. 0%; *p* = 0.007) in a population of progressive p-NETs [79]. The most frequent grade 3/4 adverse events with sunitinib were neutropenia (12%), hypertension (10%) and fatigue (5%) [79].

Sorafenib, another TKI, showed interesting results in the treatment of chemoresistant metastatic enteropancreatic NETs. Partial responses (PR) were observed in 22% of patients with p-NETs. [80].

Pazopanib, another TKI, was evaluated in a phase-2 study. Pazopanib was administered to 52 patients with advanced low-grade NETs who received pazopanib (800 mg/day orally) and octreotide. In the p-NETs group, five patients had PR (RECIST), and the median PFS was 11.7 months [81].

Bevacizumab is a monoclonal antibody directed against VEGF. Combination therapies with bevacizumab have also been investigated in p-NETs. In 34 patients with advanced NETs, the combination of bevacizumab and temozolomide showed a 33% response rate in 15 patients with p-NETs [82]. In the non-randomized BETTER trial, bevacizumab associated with 5-FU and SPTZ showed a significant disease control rate (56% PR and 44% stabilization) in patients with progressive metastatic p-NETs [83]. The ongoing randomized BETTER2 trial will evaluate the effect of bevacizumab combined with chemotherapy in the first-line setting (NCT03351296).

Finally, lenvatinib, a multikinase inhibitor, has shown promising results in a phase II trial in patients with advanced p-NETs. The overall response rate was 44.2% in the pre-treated p-NET population [84].

#### 3.4.2. mTOR Inhibitors

mTOR is an intracellular protein kinase that participates in PI3K/Akt signal transduction. Everolimus had shown promising antitumor activity in two phase II trials involving p-NETs [85,86]. Later, in a double-blind phase III trial, everolimus reduced the estimated risk of progression or death by 65% and significantly increased the median PFS from 5.4 to 11 months (HR, 0.35; *p* < 0.001) in patients with low- or intermediate-grade progressive p-NETs [87]. The estimates of 18-month PFS were 34% for patients treated with everolimus compared to 9% for patients receiving a placebo [87]. Given those results the rapamycin analog everolimus is approved for the treatment of p-NETs.

### 3.5. Peptide Receptor Radionuclide Therapy (PRRT)

The efficacy of PRRT (Lutetium−177 (177Lu)-Dotatate) combined with SSA was proven in the prospective phase III NETTER-1 trial in 229 patients with advanced well-differentiated midgut NET [88]. Positive results with PRRT were also found in a retrospective study for advanced G1/G2 p-NETs [89]. Indeed, there was an overall response of 55% and a median PFS of 30 months in a population of 133 advanced p-NETs. Overall, PRRT can be proposed in the second-line setting for advanced p-NETs.

### 3.6. External Radiotherapy

Several retrospective studies have suggested that external beam radiotherapy and stereotactic body radiotherapy is well tolerated and showed encouraging activity with a good local control on the primary tumors [90,91] but also in metastatic locations of the disease [92,93] and should be considered for further investigation.

## 4. Surgical Management of Advanced p-NETs

Surgical treatment of advanced and/or metastatic NETs remains controversial. Concerning p-NETs, surgery should always be included in a multimodal approach among different treatments available, including somatostatin analogs, PRRT, targeted therapies (everolimus and sunitinib), and chemotherapy [94]. Even if the impact of liver metastases (LM) resection on survival remains unclear, the resection of both LM and primary tumor should be discussed, especially for well differentiated p-NETs if R0 resection could be achieved [95,96,97]. In order to control hormonal symptoms, some patients presenting with functioning tumors will be considered for cytoreductive surgical procedures. In the meantime, if the risk–benefit balance seems favorable to surgery regarding patient comorbidities, borderline equivalent or even locally advanced patients will be candidates for pancreatic resection extended to adjacent organs [98]. The ENETS guidelines’ criteria to recommend surgical treatment of LM coming from digestive NETs include the differentiation of the lesions (Ki67 < 5%), the absence of extra-abdominal disease or peritoneal carcinomatosis and the absence of right cardiac insufficiency [99]. Surgery should also be considered in selected cases of symptomatic oligo-metastatic extra-hepatic disease [100].

### 4.1. Surgery of Liver Metastases

At diagnosis, curative resection cannot be achieved in more than 80% of patients with LM [101]. If surgical cytoreduction of at least 90% of LMs is possible using either surgery or percutaneous ablative procedures, those procedures should be proposed [102,103,104] and are associated with an improvement in both functional and non-functional symptoms in 90 to 100% of patients and 5-years and 10-years overall survival around 46–86% and 35–79%, respectively [105,106,107,108,109,110,111,112,113,114,115,116]. For LM < 2 cm, radiofrequency ablation is safe and provides effective control of symptoms with a low recurrence rate. [117,118]. Elias et al. reported that preoperative imaging is not sufficiently sensitive and misses half of the liver metastasis [119] but new imaging techniques such as gallium TEP/CT will probably provide better results in preoperative patient screening.

### 4.2. Management of Primary Tumor

Despite the high risk of recurrence, combined pancreatic and metastatic treatment remains the best option whenever possible to obtain long-term survival [120,121]. However, resection of pancreatic tumors in the presence of unresectable LM should be selectively considered in patients with symptomatic non-functioning p-NETs [122,123] and in low-risk patients with life-threatening symptoms [124]. Regarding p-NETs, in a systematic review including one prospective study and two retrospective studies, a greater 5-year survival rate was observed in patients who had their primary tumor resected [125,126,127,128].

### 4.3. Liver-Directed Therapies

In the metastatic setting, liver-directed intra-arterial therapies can be performed alone or in combination with surgery. These therapies include trans-arterial embolization, trans-arterial chemoembolization (with doxorubicin or streptozotocin), as well as radioembolization. Although the data mostly comes from retrospective small-sample sized studies, liver-directed therapies have shown good clinical and morphological responses for well-differentiated G1 and G2 NET, especially when the liver burden is important or in the presence of a secretory syndrome [129,130]. To date, data are still lacking to recommend one intra-arterial therapy over another, but there seems to be a trend of superiority for chemoembolization in p-NETs [130].

### 4.4. Minimally Invasive Approach

The role of minimally invasive hepatic resection in p-NETs with liver metastases follows the same principles as in open resection [131,132,133,134]. The minimally invasive approach reduces the length of stay, allows faster postoperative recovery and easier iterative hepatectomy if necessary [135].

From a scientific point of view, only a few series focused on laparoscopic resection for NET with liver metastases showing similar outcomes in the laparoscopic group compared to the open group [136,137].

Detectability remains an important matter of concern when treating metastatic NETs with minimally invasive surgery. In the future, intraoperative contrast-enhanced ultrasound imaging could be of significant assistance [138].

### 4.5. Liver Transplantation

A review of the European Liver Transplant Registry (ELTR) data showed that transplant experience in NET patients is limited [139,140,141,142]. Some expert centers have suggested the use of strict inclusion criteria to considerer liver transplantation in metastatic NETs such as (i) well-differentiated NETs (Ki-67 < 5%); (ii) portosystemic tumor drainage; (iii) young patients < 55 years; (iv) stable disease for at least 6 months; (v) pre-transplant R0 primary tumor resection; (vi) hepatic tumor involvement < 50% of the liver volume; and (vii) absence of extra-hepatic disease [143]. In the European series published by Le Treut et al. [144], 17% of patients died from early or late complications of liver transplantation and the 5-year OS rate was 52% with a disease-specific survival rate of 30%. These results suggest that liver transplantation should be considered an option in highly selected patients with liver metastases from p-NETs that are not accessible to other treatments.

## 5. Conclusions

P-NETs are a heterogeneous type of pancreatic tumor associated with good prognosis compared to pancreatic adenocarcinoma. Due to the rarity of the disease, many questions are still unanswered regarding the optimal treatment strategy for p-NETs. Indeed, comparative studies are scarce in the literature. In the case of localized disease, minimally invasive surgery, including parenchymal sparing procedure, should be preferred. Laparoscopic surgery provides a real advantage while preserving surgical oncological principles and, in the future, robotic-assisted surgery could overcome the laparoscopic approach. Functioning p-NETs are usually detected early because of specific symptoms. On the contrary, in the case of a non-functioning P-NETs a high range of patients show synchronous metastases at diagnosis mainly located in the liver. However, whenever possible, surgical resection should be attempted in combination with liver-directed therapy. In the setting of aggressive unresectable disease, combining therapies with SSA or cytotoxic chemotherapy should be considered first. Sunitinib, everolimus and PRRT also play a relevant role in the second-line treatment of p-NETs. Early clinical trial results support the idea that small-molecule-targeted therapies are efficient in the treatment of p-NETs. In the future, biomarker identification should predict responses to specific therapies, which can be used to guide the selection of treatments.

**Figure 1:**
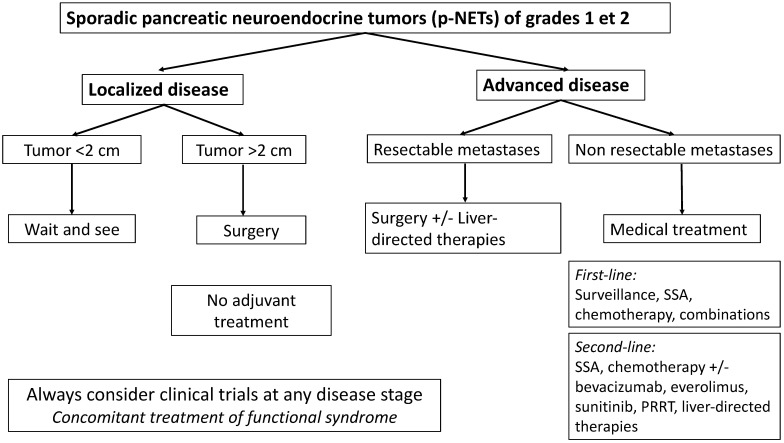
Proposed therapeutic algorithm for sporadic, well-differentiated grade 1 and 2 pancreatic neuroendocrine tumors (p-NETs). SSA: somatostatine analogues, PRRT: Peptide receptor radionucleide therapy.

We propose the following therapeutic algorithm for the management of p-NET G1/G1 (Figure 1).

## Figures and Tables

**Table 1:** The 2019 World Health Organization (WHO) classification for neuroendocrine neoplasms (NEN) of the digestive tract.

Well-Differentiated	Ki-67 Index (%)
NET ^1^ G1 (low-grade)	<3
NET G2 (intermediate-grade)	3–20
NET G3 (high-grade)	>20
**Poorly differentiated NEN**	
NEC ^2^ G3 Small-cell type or Large-cell type	>20
Mixed neuroendocrine-nonneuroendocrine neoplasm (MiNEN)	

^1^ NET: neuroendocrine tumor, ^2^ NEC: neuroendocrine carcinoma.
